# Current Technologies in Snake Venom Analysis and Applications

**DOI:** 10.3390/toxins16110458

**Published:** 2024-10-25

**Authors:** Henrique Roman-Ramos, Paulo Lee Ho

**Affiliations:** 1 Laboratório de Biotecnologia, Programa de Pós-Graduação em Medicina, Universidade Nove de Julho (UNINOVE), São Paulo 01504-001, SP, Brazil; ramoshr@uni9.pro.br; 2 Centro Bioindustrial, Instituto Butantan, São Paulo 05503-900, SP, Brazil

**Keywords:** snake venom, proteomics, genomics, transcriptomics, bioinformatics, mRNAs, synthetic biology, venomics, toxin evolution, antivenom, therapeutic potential

## Abstract

This comprehensive review explores the cutting-edge advancements in snake venom research, focusing on the integration of proteomics, genomics, transcriptomics, and bioinformatics. Highlighting the transformative impact of these technologies, the review delves into the genetic and ecological factors driving venom evolution, the complex molecular composition of venoms, and the regulatory mechanisms underlying toxin production. The application of synthetic biology and multi-omics approaches, collectively known as venomics, has revolutionized the field, providing deeper insights into venom function and its therapeutic potential. Despite significant progress, challenges such as the functional characterization of toxins and the development of cost-effective antivenoms remain. This review also discusses the future directions of venom research, emphasizing the need for interdisciplinary collaborations and new technologies (mRNAs, cryo-electron microscopy for structural determinations of toxin complexes, synthetic biology, and other technologies) to fully harness the biomedical potential of venoms and toxins from snakes and other animals.

## 1. Introduction

Snake venom has long been a subject of fascination and research due to its complex composition and potent biological activities. Historically, the study of snake venom has provided critical insights into pharmacology [1,2,3], toxinology [4], and the development of life-saving antivenoms [5,6,7]. Traditional techniques such as protein purification and basic biochemical assays have laid the groundwork for understanding venom components [8,9]. However, these methods often fell short in providing a comprehensive understanding of venom complexity and its full pharmacological potential.

**Figure 1:**
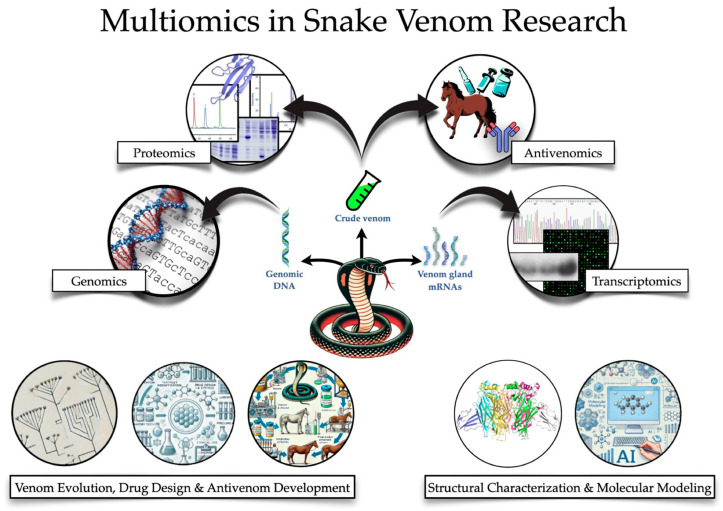
Snake venom characterization. Crude venom can be characterized through venomics, which includes protein identification by proteomic, transcriptomic, or genomic studies. Antivenomic studies can be conducted to validate the efficacy of antiserum against both homologous and heterologous venoms. These technologies enhance our understanding of the structure and function of toxins, potentially leading to the development of new drugs, antidotes, and insights into the evolution of venom toxins.

Technological advancements in proteomics [10,11,12], genomics [11,13], structural biology [14], and bioinformatics [11,15] have revolutionized the field, allowing scientists to delve deeper into the molecular intricacies of snake venoms. Proteomics, for instance, enables the detailed identification and quantification of venom proteins, uncovering intricate toxin compositions that vary among species and even individual snakes [16,17]. Additionally, genomic techniques, including next-generation sequencing (NGS) and transcriptomic analysis, have illuminated the genetic basis of venom composition, production, and evolution, providing a broader context for proteomic data [18,19,20,21,22] (Figure 1).

Cryo-electron microscopy (Cryo-EM) has revolutionized structural biology allowing the structure determinations of protein complexes [23,24,25,26,27]. Bioinformatics and in silico tools have further propelled venom research. Advanced protein structure prediction tools like AlphaFold2 [28], ColabFold [29], and Rosetta [30] have significantly improved our ability to model and predict venom protein structures, enhancing our understanding of their functional roles and interactions with molecular targets [31]. Furthermore, molecular docking studies have facilitated the virtual screening of venom components for potential therapeutic applications, including identifying venom peptides with anti-SARS-CoV-2 activities [32].

Despite these advancements, challenges persist. The complexity of venom proteomes, the requirement for high-cost equipment, and the need for specialized expertise remain significant hurdles. Moreover, accurately quantifying venom components and predicting their biological activities in vivo necessitate ongoing methodological refinements and interdisciplinary collaborations.

This review comprehensively overviews the modern tools and techniques employed in snake venom research. It examines applications in proteomics, genomics, structural determinations, mRNA, synthetic biology, and bioinformatics, highlighting how these methods overcome previous limitations and advance our understanding of venom biology. Additionally, it discusses persistent challenges and proposes future research directions to unlock the full potential of snake venoms in biomedicine and envenomation therapies.

## 2. Genomic Insights into Snake Venom Evolution

Snake venoms are complex mixtures of bioactive molecules, predominantly proteins and peptides, which have evolved under strong natural selection pressures for prey capture and defense. Genomic research, in particular, has revolutionized our understanding of venom evolution, unveiling the intricate genetic mechanisms underlying venom production, diversification, and adaptive variation [13,33]. Genomic tools have also contributed significantly to the development of next-generation antivenoms and therapeutic applications [34,35,36,37,38,39]. In this section, we explore how genomics provides essential insights into venom evolution, gene duplication, and toxin variability, while also discussing how these technologies are shaping venom research and practical applications.

### 2.1. Genomic Mechanisms Driving Venom Evolution

Gene duplication and positive selection are fundamental forces in snake venom evolution, driving the diversification and neofunctionalization of toxin gene families [21,36,40,41,42]. Duplicated genes provide the raw material for evolutionary innovation, while positive selection promotes mutations that enhance venom potency, improving prey capture and defense mechanisms. These processes have profoundly shaped toxin families such as phospholipase A_2_s (svPLA_2_) and snake venom metalloproteinases (SVMPs) across multiple snake lineages [40,43].

Alongside these mechanisms, alternative splicing (AS) and recombination add further layers of diversity. AS enables a single gene to generate multiple distinct mRNA transcripts, expanding functional diversity beyond gene duplication. Recombination also contributes by producing structural variations in proteins, leading to novel venom phenotypes [44,45,46].

The habu snake (*Protobothrops flavoviridis*) exemplifies the role of AS in venom evolution. AS generates multiple isoforms from the same gene within families such as SVMPs, serine proteases (SVSPs), and vascular endothelial growth factors (VEGFs), enhancing venom complexity [47]. In the SVMP family, splicing produces metalloproteinase variants tailored to ecological needs, such as hemorrhage induction or tissue degradation. Similarly, the SVSP and VEGF families express diverse isoforms that influence venom properties, including fibrinolysis and blood coagulation [47]. Trans-splicing—recombining mRNA from neighboring genes—further expands the venom’s functional repertoire, showcasing the intricate regulation underlying venom production [48].

These molecular insights are particularly valuable in understanding regional venom variation, which can complicate treatment. Traditional antivenoms are often polyvalent, aiming to neutralize a broad range of toxins. However, by identifying specific isoforms expressed in local populations, therapies could become more precise, reducing the likelihood of off-target effects and improving patient outcomes [47,48].

Molecular and phylogenetic studies also show that PLA_2_ isozyme genes have evolved rapidly in species such as *Sistrurus* rattlesnakes, driven by dietary shifts [40,49]. A similar pattern is observed in three-finger toxins (3FTxs), which evolved to target nicotinic acetylcholine receptors (nAChRs) with remarkable specificity, exemplifying how adaptive pressures drive innovation [50]. The structural diversity within venom proteins further demonstrates how positive selection fosters rapid evolution [5]. Molecular clock analyses suggest that many toxin families originated millions of years ago through ancestral gene duplications [46,47].

Phylogenomic approaches offer further insights into the evolution of venom systems across snake lineages [37,51]. Comparative studies reveal convergent evolution, where unrelated species develop similar venom components to adapt to shared ecological niches [52,53]. For example, the parallel evolution of SVMPs and PLA_2_s across snake families illustrates how gene co-option and positive selection shape functionally analogous toxins [37,54,55].

The recruitment of salivary proteins into venom glands provides another layer of molecular innovation, showing how existing proteins can acquire toxic functions [56]. This adaptability is further demonstrated by the loss of toxin genes under changing environmental conditions, such as the differential expression of neurotoxic PLA_2_s in rattlesnake populations [57]. These findings highlight how venom systems remain dynamic, evolving in response to ecological pressures, and underscore the importance of tailoring antivenom development to reflect local venom profiles [58,59].

### 2.2. How Genomics Powers Venom Research and Antivenom Development

**Figure 2:**
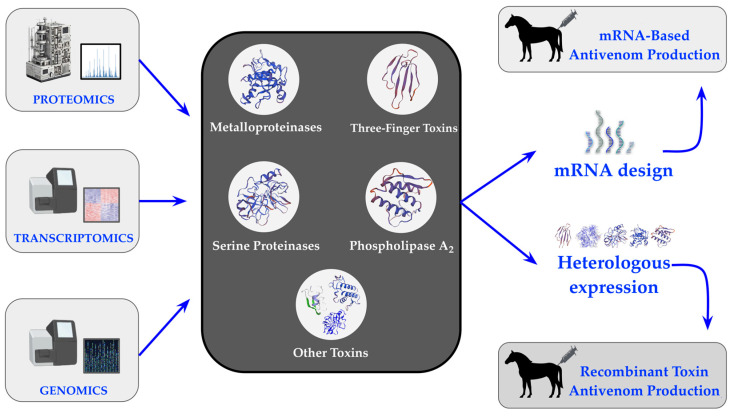
Snake venoms are complex mixtures of proteins and peptides used for defense and to subdue prey. Based on transcriptomic and proteomic studies of the venom gland and venom, the mean abundance and occurrence of 42 toxin families were identified and classified into 4 dominant, 6 secondary, 14 minor, and 18 rare protein families [38]. Phospholipase A2 (PLA2), snake venom metalloprotease (SVMP), three-finger toxins (3FTx), and snake venom serine protease (SVSP) are the four dominant toxin families. Therapy for snakebite envenomation is typically based on serum therapy obtained from immunized animals, such as horses, using crude venom. Alternatively, it may be possible to produce hyperimmune antivenom by immunizing animals with recombinant toxins or mRNA formulated in lipid nanoparticles, similar to mRNA vaccines.

The advent of high-throughput sequencing has transformed venom research by enabling the rapid identification of venom-related genes and their regulatory networks [33,34,35]. Technologies such as VenomCap, developed by Travers et al., facilitate exon-capture sequencing, accelerating the mapping of venom gene repertoires across diverse snake species [60]. However, a key challenge lies in effectively interpreting the large datasets generated by these technologies. Without robust bioinformatics tools, fragmented genomic data may limit the utility of high-throughput sequencing for both evolutionary research and practical applications [38,61,62] (Figure 2).

Comparative genomic studies reveal that venom genes often cluster with non-venom paralogs, supporting the role of gene co-option in venom evolution [37,57]. This finding presents both opportunities and challenges: while gene co-option promotes venom diversity, it also complicates efforts to distinguish functional toxins from non-venom genes, which poses difficulties for targeted antivenom development. Additionally, epigenomic mechanisms, such as chromatin loops, modulate gene expression, helping venom glands control toxin production [35,63,64]. However, the dynamic nature of venom regulation raises questions about whether environmental conditions or life stages could induce unpredictable shifts in venom composition, making the development of standardized treatment approaches more complex.

Genomic insights are revolutionizing antivenom production by enabling the design of tailored antivenoms that target specific venom components. Traditional antivenoms often struggle with regional and intraspecific venom variability, as exemplified by the variability in crotamine expression across *Crotalus durissus* populations [65,66,67]. This limitation highlights a critical drawback of polyvalent antivenoms, which are not always effective across all venom variants within a species [68,69]. With genomics, researchers can identify clinically relevant toxin isoforms, allowing the creation of region-specific antivenoms tailored to the venom compositions of local snake populations. This precision improves treatment efficacy and reduces off-target effects, which is especially critical in tropical regions where inconsistent venom profiles complicate treatment and snakebite envenoming remains a major public health concern [39,61,68].

The use of recombinant toxins and synthetic antibodies has further revolutionized antivenom production, reducing dependence on crude venom extractions and enhancing consistency in formulations [48,58]. However, these innovations raise concerns about cost and accessibility, especially in low-resource settings where snakebites are most prevalent. Without coordinated efforts, these advances may remain confined to research-intensive or high-income regions, limiting their global impact.

On the evolutionary front, genomics has unveiled how venom systems evolved through mechanisms like gene duplication and protein co-option, enhancing our understanding of venom’s adaptive plasticity [63]. Convergent evolution has also been observed, with unrelated snake species developing similar toxins to adapt to analogous ecological niches [39,69]. This convergence offers both opportunities and challenges for antivenom development: while it suggests the potential for broad-spectrum antivenoms, it also requires precise molecular characterization to avoid neutralizing essential non-venom proteins [48,58,67].

In summary, genomic technologies have fundamentally reshaped both venom research and antivenom development. However, challenges remain, including the need for advanced bioinformatics tools to manage complex datasets, ensuring global access to new antivenom strategies, and accounting for environmental factors that influence venom expression. By integrating evolutionary insights with practical medical applications, these technologies hold great promise for improving antivenom therapies and reducing fatalities, particularly in underserved regions where snakebite envenoming is a persistent health threat [58,68].

### 2.3. Regulatory Networks and Gene Expression Control in Venom Systems

The expression of venom genes is regulated by complex genomic networks involving transcription factors, enhancers, and non-coding RNAs [19,35,70]. For example, the transcription factor Sp1 is upregulated in elapid venom glands following milking, promoting the expression of key toxins [19]. Additionally, microRNAs play a role in post-transcriptional regulation, modulating the expression of toxin genes across life stages [63,70].

Epigenetic regulation further adds to the complexity of venom gene expression. Topologically associated domains (TADs) and other chromatin structures influence which genes are expressed in response to environmental or physiological stimuli [35,64]. These regulatory mechanisms enable venom glands to adjust their toxin output based on prey availability and ecological pressures [53,71].

### 2.4. Future Directions in Genomics and Venom Research

The future of venom research lies in the continued integration of genomic data with other ‘omics’ technologies, such as transcriptomics and proteomics [34,70]. Long-read sequencing technologies and methylation studies will offer further insights into regulatory elements controlling venom genes. Personalized antivenom strategies may also become a reality, with formulations designed for the specific venom profile of the snake involved in an envenomation [65,68].

Genomic databases will play a key role in identifying novel toxin candidates for pharmaceutical development, leveraging snake venoms for drug discovery and biotechnology applications [48,72]. As genomic tools become more accessible, they will continue to transform snakebite management and provide new opportunities for exploring the therapeutic potential of venom components [48,54].

## 3. Synthetic Biology

Synthetic biology, when applied to venom research, holds great promise for expanding our understanding and exploitation of venom components, either for therapeutic purposes or for studying structure–function relationships. The integration of synthetic biology techniques with systems biology approaches has provided new insights into the complexities of venom composition, function, and its application in novel therapies [73]. These advancements are particularly relevant in the context of snakebite envenomation, a significant global health concern that remains challenging to address through conventional methods [74].

This section builds upon these recent advancements, delving into key areas of progress, such as the recombinant expression of venom toxins using heterologous systems, the development of synthetic antibodies and antivenoms, and genetic immunization strategies. It will also explore the potential of multiepitope DNA constructs, and the emerging role of mRNA therapeutics in antivenom development. Collectively, these innovations illustrate how synthetic biology is transforming venom research and facilitating the creation of more effective and accessible snakebite treatments.

### 3.1. Production of Venom Toxins Using Heterologous Expression Systems

The production of venom toxins using heterologous expression systems represents a critical development in venom research, providing researchers with tools to explore the structure–function relationships of these molecules in unprecedented detail [75]. One of the early studies that highlighted this was conducted by André Menez (*in memoriam*), who focused on three-finger toxins and their interaction with nicotinic receptors [76,77,78,79]. Menez demonstrated that specific residues in these toxins are key to their biological activity, showing how recombinant proteins can be used as precise tools in such investigations.

Building on these foundational insights, the choice of an appropriate heterologous expression system becomes a key consideration, as it directly influences both the quality and quantity of the toxin produced. Different expression systems offer distinct advantages and limitations, which must be carefully evaluated to meet the specific requirements of the toxin being studied.

#### 3.1.1. Bacterial Systems

Bacterial systems such as *Escherichia coli* are highly efficient for expressing small, cysteine-free toxins and offer advantages for large-scale recombinant protein production and high-throughput screening due to their rapid growth rates, ease of genetic manipulation, and low production costs. Nevertheless, these systems often encounter difficulties in forming correct disulfide bonds in more complex proteins and are limited by their inability to perform complex post-translational modifications (PTMs), which are crucial for the functionality of many eukaryotic proteins.

To address issues like protein misfolding and aggregation, strategies such as the co-expression of molecular chaperones (e.g., GroEL/GroES), and the use of specialized bacterial strains like SHuffle^®^ (New England Biolabs, Ipswich, UK), and Origami™ (Novagen, Madison, WI, USA) have been employed [80,81,82,83]. Both strains are engineered with mutations in the *trxB* and *gor* genes, which disable the reducing environment of the cytoxplasm, thus favoring the formation of disulfide bonds. Additionally, SHuffle^®^ strains express DsbC, a disulfide bond isomerase that works as a folding catalyst in the cytoplasm, further aiding in the correct arrangement of these bonds [84].

One potential use of *E. coli* Origami™ strains for the expression of a synthetic short-chain consensus α-neurotoxin (ScNtx) was demonstrated by de la Rosa et al., who successfully achieved protein yields of 1.5 mg/L in culture medium. This approach enabled the expression of the protein in a soluble form, preserving functional motifs similar to those of native toxins [85]. The recombinant production of ScNtx was later optimized, enhancing both yield and solubility, which facilitated structural studies. These studies revealed that ScNtx binds to muscle-type nicotinic acetylcholine receptors (nAChRs) via a distinct interaction involving loops C and F, differing from the binding mechanism observed in long-chain α-neurotoxins, as shown by cryo-electron microscopy analysis [27].

With regard to SHuffle^®^ strains, although some studies have reported toxin expression in the form of IB [86,87], this strain has proven effective for the soluble expression of disulfide-rich proteins such as insulin-like growth factors [88] and antibodies [89]. SHuffle^®^’s ability to enhance oxidative folding, particularly through the co-expression of DsbC isomerase, provides a valuable solution when correct disulfide bond formation is critical.

The use of fusion tags—such as Glutathione S-transferase (GST), Small Ubiquitin-like Modifier (SUMO), or Maltose-binding Protein (MBP)—has also shown promise in improving the solubility of recombinant toxins by promoting proper folding and preventing aggregation. These tags can be cleaved off post-expression to restore the native structure and activity of the toxins [90]. Alternatively, lowering the expression temperature to 16 °C has been shown to reduce the formation of inclusion bodies (IBs) and increase the yield of soluble proteins, including toxins. This strategy slows down the rate of protein synthesis, allowing more time for proper folding and disulfide bond formation. This method has been particularly useful in expressing disulfide-rich proteins such as svPLA_2_ and other venom-derived toxins [27,86,87].

Despite these efforts, recombinant expression in bacterial systems very often results in the formation of inclusion bodies (IBs)—aggregates of misfolded proteins. While IBs are typically seen as a drawback, they can offer advantages when expressing cytotoxic proteins, such as svPLA_2_. In this context, IBs allow for the production of large quantities of these toxins in an inactive and concentrated form, protecting the host cells from their harmful effects. Once harvested, these proteins can be refolded in vitro to ensure proper folding and disulfide bond formation. In fact, the production of recombinant toxins in inclusion bodies, followed by in vitro refolding, has been shown to be effective in achieving correct folding and disulfide bond formation [91].

#### 3.1.2. Yeast Systems

Yeast systems, particularly *Pichia pastoris*, offer a valuable platform for recombinant toxin expression, especially when more complex PTMs are necessary [92]. Unlike bacterial systems, yeast cells can perform essential eukaryotic PTMs, such as glycosylation and proper disulfide bond formation, which are often crucial for the biological activity and stability of venom toxins. This ability makes yeast systems particularly advantageous for expressing more complex proteins. Moreover, yeast expression systems retain key benefits such as rapid growth and low production costs, similar to bacterial systems, while providing enhanced capabilities for handling proteins that require intricate modifications.

*Pichia pastoris* has become widely used for expressing venom toxins rich in disulfide bonds, including snake venom serine proteinases (SVMPs) [93,94]. This yeast species is known for its high-level expression capability, and its ability to secrete proteins into the culture medium simplifies the purification of recombinant toxins. Notably, *P. pastoris* has been successfully used to produce bioactive snake venom metalloproteinase (SVMP) inhibitors [95], as well as other disulfide-rich venom components such as svPLA_2_ [96].

One of the key advantages of yeast systems is their ability to secrete recombinant proteins, reducing the need for cell lysis and minimizing protein aggregation [97]. Additionally, yeast cells can co-express molecular chaperones, such as BiP and Pdi1p, which assist in proper protein folding and disulfide bond formation—functions that are comparable to the role of DsbC in bacterial systems [98]. This combination of secretion and molecular chaperone support makes yeast systems particularly effective for producing bioactive proteins, further enhancing their suitability for expressing venom toxins that require intricate folding, and overcoming many of the limitations associated with bacterial expression systems.

#### 3.1.3. Insect and Mammalian Cell Expression Systems

Insect and mammalian cell expression systems offer complementary strengths when it comes to the production of recombinant proteins, particularly animal venom toxins. Insect cells, through the baculovirus expression system (BEVS), are ideal for high-throughput, cost-effective production of functional toxins. These cells can fold complex cysteine-rich peptides and perform PTMs like glycosylation and acetylation, which are essential for the functionality of many venom proteins. Due to their evolutionary closeness to arthropods, insect cells are particularly suitable for expressing toxins from arachnids, insects, and other similar species, where proper folding and function are crucial [99]. For example, toxins such as the Pctx1 spider toxin have been successfully expressed in insect cells without the need for in vitro refolding [100].

Conversely, mammalian cells are the preferred system for producing proteins that require even more intricate PTMs and glycosylation patterns. While insect cells can glycosylate proteins, the structure of the glycans is often simpler compared to those produced by mammalian cells. Mammalian systems like Chinese Hamster Ovary (CHO) cells or Human Embryonic Kidney 293 (HEK293) cells are capable of producing fully human-like glycoproteins with complex sialylation, which is often critical for the stability and functionality of therapeutic proteins [101,102]. These systems are particularly useful for the production of complex venom proteins that require precise glycosylation and folding, such as certain snake venom toxins that affect human receptors [103].

Both systems have their challenges. While insect cell systems like BEVS offer versatility and high yields, they are slower to set up and require complex facilities for the production of recombinant baculoviruses. Conversely, mammalian systems, although capable of producing proteins that closely resemble their native forms, are more expensive and yield lower quantities of protein compared to insect or microbial systems. This makes mammalian cells better suited for the production of highly specialized proteins, particularly when precise glycosylation and folding are needed, as is often the case for certain snake venom toxins, such as rhodocytin [104] and ecarin [105].

Looking forward, advances in both expression systems are bridging the gap between cost and functionality. Insect cells are increasingly being engineered to produce human-like glycosylation patterns, while mammalian cells are benefiting from innovations in gene editing and culture techniques that improve yields and reduce costs. Both systems remain crucial for venom toxin research and biopharmaceutical applications, with insect cells offering a more accessible platform for routine toxin production and mammalian cells providing the gold standard for producing complex, highly modified proteins [102].

However, the cost of insect cell systems is higher than bacterial systems, though still generally lower than mammalian cell cultures. Additionally, while they can handle more complex proteins, the level of post-translational fidelity, particularly for glycosylation, may still be insufficient for certain therapeutic applications. This limitation has prompted researchers to develop engineered insect cell lines with more human-like glycosylation patterns [106]. Despite these challenges, insect cells remain a versatile and efficient system for producing proteins that require moderate complexity in their folding and post-translational modifications.

#### 3.1.4. Cell-Free Systems

Cell-Free Systems (CFSs) provide a rapid and flexible alternative to traditional cellular expression systems, eliminating the reliance on living cells to produce proteins. These systems are particularly advantageous in scenarios where time constraints or the expression of cytotoxic proteins, such as certain venom toxins, make cell-based systems impractical [107]. Derived from cellular extracts that retain the essential components for transcription, translation, and some post-translational modifications, CFSs allow the direct synthesis of proteins from DNA or mRNA templates, bypassing the regulatory mechanisms of living cells that often hinder the production of toxic or unstable proteins [108]. This makes CFSs highly suitable for high-throughput applications, particularly when expressing proteins that tend to form inclusion bodies or are difficult to produce using conventional expression systems. The scalability and efficiency of these systems have been significantly improved in recent years, enabling their application in structural genomics and proteomics, as well as in the production of complex proteins that require specific folding or modifications [107,108].

One of the significant benefits of CFSs is their capacity to incorporate non-standard amino acids, facilitate disulfide bond formation, and conduct essential post-translational modifications. These features are crucial for the correct folding and activity of many complex toxins, including those derived from snake venom. CFSs, particularly defined systems like the PURE system, provide a controlled environment for protein synthesis, enabling the precise manipulation of reaction conditions to optimize the expression of complex proteins requiring specific folding and modifications [109]. This is especially important for producing disulfide-rich peptides, such as α-neurotoxins, which rely on accurate disulfide bond formation for their biological function. Additionally, the open nature of CFSs allows for direct manipulation of the protein production environment, ensuring optimal conditions for challenging proteins, like venom toxins, that are difficult to express in traditional systems [110]. The ability to modify and optimize these systems makes them highly effective for expressing proteins that require intricate folding, which is often not achievable using cell-based methods.

Despite their flexibility, CFSs face limitations in scalability and cost. Large-scale production remains challenging, as bacterial systems such as *E. coli* continue to be the most cost-effective option for producing large quantities of recombinant proteins [111]. These bacterial systems are ideal for industrial-scale applications due to their ease of scalability and low production costs, particularly when significant amounts of protein are required [112]. However, CFSs excel in smaller-scale applications, such as the rapid prototyping of complex venom toxins for structural and functional studies. CFSs allow the efficient synthesis of proteins that are difficult to produce in living systems due to toxicity or folding complexity, such as venom metalloproteinases and other disulfide-rich toxins [113]. In venom research, for example, CFSs have been employed to produce small quantities of toxins, enabling rapid testing of their structure and function without the need for large-scale production, making them invaluable for experimental assays where precision and speed are crucial [112].

Ultimately, the choice between bacterial systems and CFSs depends on the specific requirements of the toxin being studied, including factors such as the need for post-translational modifications, scalability, and production speed. While CFSs offer distinct advantages for producing complex and cytotoxic proteins, their limitations in large-scale production and the cost remain challenging. However, ongoing advancements are addressing these issues, as seen in venom research where CFSs have been employed to synthesize venom components such as spider toxins [114]. These systems enable rapid laboratory-scale production, overcoming challenges posed by limited venom yields from small species. Despite hurdles like disulfide bond formation, improvements in system optimization and protein folding are enhancing the applicability of CFSs for venom bioprospecting, making them increasingly valuable tools in this field.

### 3.2. Phage Display and Synthetic Antivenoms

The evolution of snakebite treatment from animal-derived antivenoms to recombinant and synthetic alternatives represents a major step forward in medical biotechnology. Although traditional equine-derived antivenoms have been lifesaving for over a century, they face numerous limitations, including batch variability, high production costs, and adverse reactions like serum sickness. These challenges have driven the search for alternatives such as monoclonal antibodies, synthetic antibody libraries, and phage display technology. However, despite their potential, several technical and economic barriers must be addressed before these innovations can become widely available in regions most affected by snakebite envenoming.

One key technological advance in this field has been the use of phage display to develop synthetic antibodies. In one study, researchers demonstrated the effectiveness of this technique by developing a panel of synthetic antibodies capable of neutralizing α-cobratoxin, a potent α-neurotoxin from *Naja kaouthia* [115]. These antibodies were shown to block the binding of the toxin to nicotinic acetylcholine receptors (nAChRs) and prevent its curare-like effects, achieving sub-nanomolar affinities. This highlights the precision and potential efficacy of phage display.

Nonetheless, translating the in vitro success of phage display into effective in vivo treatments remains a significant challenge. Venoms are complex mixtures, and neutralizing just one toxin may not be sufficient to mitigate the systemic effects of envenoming. Another study demonstrated the high effectiveness of phage-display-selected antibodies in neutralizing α-cobratoxin in vitro but stressed that further testing in animal models is required to confirm their efficacy in complex biological systems [115]. This gap between in vitro and in vivo performance is a key hurdle in advancing phage display-derived antibodies into clinical use.

The ability of phage display to generate antibodies against poorly immunogenic toxins, such as svPLA_2_ and three-finger toxins (3FTx), is a major advantage. Evidence suggests that these venom components are notoriously difficult to neutralize using traditional polyclonal antibodies due to their low immunogenicity and small size [116]. This specificity offers the opportunity to significantly improve the effectiveness of antivenoms. However, the downside is that highly specific monoclonal antibodies may lack the broad-spectrum activity needed to neutralize the full range of toxins present in whole venoms, making it difficult to create a universally effective antivenom. Addressing this issue will require balancing the need for specificity with broader efficacy against diverse venom toxins.

To overcome this limitation, the integration of phage display with other antibody discovery platforms has been suggested. For instance, another approach involves using plants as biofactories to produce recombinant polyclonal antibodies (pluribodies) that can target a wider range of venom components [117]. This method has shown promise in enhancing the cost-effectiveness and scalability of antivenom production while maintaining efficacy against diverse toxins. Such hybrid approaches could combine the precision of phage display with the broad-spectrum capabilities of plant-based antibody production, potentially solving many of the challenges faced by traditional antivenom therapies.

The application of phage display has also been extended to the development of broadly neutralizing antibodies (bnAbs). In recent work, a single bnAb was developed that could neutralize long-chain α-neurotoxins from several medically significant snake species, including cobras, kraits, and mambas [118]. This finding demonstrates the potential of creating universal antivenoms that target conserved toxin epitopes across different species. However, while this represents a significant advance, it has been noted that targeting only α-neurotoxins may not be sufficient to neutralize all venom effects. The inclusion of additional antibodies targeting other venom components, such as metalloproteinases and svPLA_2_s, would be essential for comprehensive venom neutralization [118].

The economic and logistical challenges of producing synthetic antivenoms at scale must also be addressed. The development of aptamers—short oligonucleotide sequences that can bind specifically to venom components—has been proposed as a cost-effective alternative [119]. Aptamers offer advantages such as lower production costs and longer shelf life compared to antibodies, making them an attractive option for low-resource settings. However, despite their promise, aptamers face limitations, particularly regarding their shorter half-life in vivo and their inability to neutralize complex venom mixtures on their own. Combining aptamers with phage display-derived antibodies could provide a more comprehensive solution, though this would also introduce additional complexity and cost in production.

Phage display, in particular, represents a transformative technology in the development of next-generation antivenoms, especially for targeting poorly immunogenic toxins. Significant challenges, however, remain in translating these advances into cost-effective, scalable treatments capable of providing broad-spectrum efficacy. By integrating phage display with other emerging technologies, such as plant-based production and aptamer-based neutralization, future antivenom development could strike the necessary balance between precision, scalability, and affordability. To ensure these treatments are widely accessible, ongoing innovation must be matched with efforts to address the financial and logistical barriers that currently limit the availability of these life-saving therapies.

### 3.3. Synthetic DNA Constructs for Antivenom Development

Genetic immunization has opened new avenues for antivenom production. By using DNA molecules to immunize animals, this approach has been shown to induce high-titer and protective antibody responses suitable for antivenom production. For instance, Harrison et al. demonstrated that DNA immunization could generate antisera that cross-react with venoms from phylogenetically distinct viper species, highlighting its potential for producing broad-spectrum antivenoms [120].

Moreover, bioinformatics tools have allowed researchers to systematically and precisely select immunoprotective sequences by identifying common antigenic epitopes from venom gland expressed sequence tag (EST) databases. This method was used to design synthetic DNA constructs containing epitopes from snake venom metalloproteinases (SVMPs), which are responsible for the main hemorrhagic effects of viper envenoming. These constructs successfully induced cross-generic antibody responses and demonstrated in vivo neutralization of venom-induced hemorrhage [121].

In a different study, transcriptomic data from the coral snake *Micrurus corallinus* were analyzed [122]. This analysis identified five major toxins (four 3FTx and one svPLA_2_) and mapped their reactive epitopes using bioinformatics and the SPOT synthesis technique. This led to the design of two multiepitope DNA sequences, which were used for genetic immunization followed by booster doses of recombinant versions of those toxins. This strategy achieved a 60% survival rate in a lethal dose neutralization assay, suggesting its potential as an alternative approach for developing specific antivenoms, without the need to maintain snakes in captivity for venom extraction [123].

### 3.4. Innovating Antivenoms with mRNA Technology

mRNA therapeutics have emerged as a promising tool for antivenom production, capitalizing on recent advancements in mRNA technologies demonstrated during the development of COVID-19 vaccines [124,125]. mRNA technology offers unique advantages over traditional approaches, including faster production, in vivo synthesis, and the ability to encode antibodies or antitoxins directly within the host [B, E]. This eliminates the need for labor-intensive venom extraction and immunization of animals, reducing costs and production time [126]. Furthermore, mRNA-based therapeutics have shown great potential for passive immunization, which has already proven effective for viral and toxin exposure treatments [127,128].

One of the critical benefits of IVT-mRNA (in vitro transcribed mRNA) lies in its ability to generate antibodies rapidly, ensuring timely responses in emergencies. This platform allows for multiplexed treatment by encoding antibodies targeting multiple venom components, addressing the complex and synergistic nature of snake venoms [126,129,130]. This flexibility enables mRNA-based antivenoms to be customized quickly, offering an efficient solution for managing the varying venom compositions of different snake species [127,128].

#### 3.4.1. Advances in Delivery Systems and Chemical Modifications

Lipid nanoparticle (LNP) technology, successfully used in COVID-19 vaccines, plays a pivotal role in the delivery of mRNA therapeutics [129]. These nanoparticles ensure efficient delivery by protecting the mRNA cargo from enzymatic degradation and facilitating its entry into target cells, ensuring sustained antibody production in vivo [125,126]. Such advancements are crucial for the development of mRNA-based antivenoms, as they allow for systemic delivery of therapeutic proteins, overcoming biological barriers [128,130].

Recent studies also highlight the significance of chemical modifications, such as the incorporation of pseudouridine and N1-methyl-pseudouridine (m1Ψ), which improve mRNA stability and reduce immunogenicity [125,130]. These modifications are essential to ensure the viability of mRNA-based antivenoms in remote and resource-limited regions, where cold-chain storage may not be feasible [129]. Poly(A) tails and optimized untranslated regions (UTRs) have further enhanced the stability and translatability of mRNAs, ensuring consistent protein expression [130].

#### 3.4.2. Scalability, Challenges, and Future Directions

One of the most significant advantages of mRNA-based antivenoms is their scalability. Unlike traditional antivenoms, which require extensive production cycles and animal-based facilities, mRNA therapeutics can be manufactured in a matter of weeks [125]. This rapid production process ensures batch manufacturing for multiple venom types, offering broad-spectrum solutions for envenomations caused by various snake species [126]. Additionally, the transient nature of mRNA expression provides a safety advantage by minimizing the risk of prolonged immunogenic effects [128].

Despite these advantages, challenges remain. The complexity of snake venoms across different species necessitates ongoing optimization of mRNA constructs to ensure broad-spectrum efficacy [124]. Future clinical trials will be essential to validate the efficacy of mRNA-based antivenoms and to establish standardized protocols for their delivery and administration [129]. Additionally, regulatory frameworks similar to those used for COVID-19 mRNA vaccines could accelerate the approval and adoption of these innovative antivenoms [125,129].

The convergence of mRNA technology and venom research marks a transformative moment in antivenom development, offering the potential to revolutionize snakebite management. By reducing reliance on animal models and aligning with sustainable biopharmaceutical practices, mRNA-based antivenoms promise a safer, more ethical, and effective solution to combat envenomations globally [127,130]. As these technologies continue to evolve, they hold the potential to save countless lives and improve public health outcomes in regions most affected by snakebites [126,128].

The integration of DNA- and mRNA-based technologies has significantly advanced our understanding of venom systems, from their genetic foundations to innovative therapeutic applications. These complementary approaches—genetic immunization using DNA and rapid, scalable protein expression through mRNA—offer unprecedented flexibility and precision in antivenom development. Together, they mark a turning point in venom research, paving the way for next-generation antivenoms that are safer, faster to produce, and more effective against diverse snake species. This convergence of breakthroughs not only transforms snakebite treatment strategies but also holds the potential to save countless lives worldwide.

## 4. Transcriptomics

Transcriptomics, the study of all RNA transcripts produced by an organism or specific tissue, has emerged as a revolutionary tool in snake venom research. It offers an in-depth understanding of the intricate mechanisms governing venom composition, regulation, and evolution. By examining the transcripts in venom glands, researchers can associate these mRNA sequences with the types and relative abundance of toxins in the venom sample. This method provides crucial insights into the pathophysiology of envenomation and, when combined with genomic studies, uncovers the complex regulatory networks that control toxin expression [37,131]. Additionally, it allows for the identification of complete transcripts expressed in the venom gland, which is essential for accurate molecular cloning and the exploration of potential biotechnological applications [132].

The combination of transcriptomics and proteomics, a powerful integrative approach known as venomics, enables researchers to bridge the gap between gene expression and protein function, providing a more comprehensive understanding of venom composition and its evolutionary implications [133,134,135]. The application of high-throughput next-generation sequencing (NGS) technologies has further revolutionized transcriptomic studies, enabling the generation of large datasets and facilitating in-depth analysis of venom gland gene expression, leading to the discovery of novel toxins and toxin families [133,135,136]. The integration of diverse ‘omics’ technologies, such as genomics, transcriptomics, and proteomics, provides a holistic view of venom systems, allowing for the exploration of various aspects of venom biology, including toxin diversity, evolution, and function [50].

### 4.1. Milestones in Transcriptomics

The evolution of transcriptomic research in snake venom has been marked by significant milestones. Early efforts, dating back to 1995, focused on sequencing a limited number of cDNAs from the venom glands of Black Mamba (*Dendroaspis polylepis*) [137] and coral snake (*Micrurus corallinus*), both belonging to the Elapidae family [138], leading to initial insights into venom gene expression. In 2002, however, a major breakthrough occurred at Instituto Butantan, where researchers created the first comprehensive set of reptilian gene sequences, culminating in an EST database that included hundreds of cDNAs from the venom gland of *Bothrops insularis* [139]. This enabled, for the first time, the identification of the most common toxin classes found in Viperidae venoms, reflecting the complex hemorrhagic effects these venoms induce in their prey.

Following these advancements, in 2006, the transcriptomic analysis of the venom gland of another medically important snake, *Lachesis muta*, was conducted [140], revealing not only variants within well-known toxin classes, such as C-type lectins (CTLs) but also molecules typically associated with toxins found in more distantly related snake groups. Notably, it included the first discovery of a three-finger toxin (3FTx) sequence in a Viperidae species—a scaffold commonly seen in Elapidae and Colubridae venoms—and an ohanin-like protein previously thought to be unique to Elapidae. Interestingly, subsequent studies would also identify ohanin in other non-elapid species, suggesting that these proteins are widespread in snake venoms and may contribute to venom-induced hyperalgesia [141,142,143,144]. Additionally, the study uncovered proteins not usually classified as toxins, such as 5’-nucleotidase and certain proteases, whose functions are analogous to known venom activities, suggesting they could be potential new toxins.

Further transcriptomic research has also explored the Duvernoy’s venom gland of rear-fanged snakes, offering additional insights into venom complexity. For example, the transcriptomic analysis of *Philodryas olfersii* not only revealed typical toxins but also identified an unusual C-type lectin (CTL) with a distinct evolutionary background, suggesting a more complex venom profile than previously thought [145]. Moreover, the presence of serine protease and metalloprotease transcripts in the venom gland explains why envenomation by this snake can be treated with anti-bothropic antisera [146,147]. This information can, therefore, guide and provide a rationale for clinical therapeutics.

Similarly, in *Thamnodynastes strigatus*, the study identified a unique composition of matrix metalloproteinases (MMPs) and other novel proteins that play crucial roles in venom-induced tissue damage, highlighting the adaptive significance of these toxins in prey immobilization [148]. Likewise, research on the tribe Pseudoboini, including *Phalotris mertensi,* provided insights into the evolutionary trends of venom toxins. This research emphasized the use of ‘omics’ approaches to uncover novel components and their adaptive roles, while also demonstrating the independent recruitment of svPLA_2_s—particularly the svPLA_2_-IIE subtype—underscoring the convergent evolution of venom components within this group [149,150].

In *Imantodes cenchoa*, the analysis showed remarkable conservation of venom phenotypes, particularly related to its specialization in lizard prey, offering a clear example of how venom composition is shaped by ecological factors [151]. Moreover, studies on various colubrid species revealed a diverse array of venom components, ranging from traditional toxic proteins to unusual enzymes, emphasizing the complexity and versatility of colubrid venoms [152]. In *Conophis lineatus*, transcriptomic data provided a comprehensive view of the venom’s toxic potential, revealing a mixture of neurotoxins and other bioactive molecules that contribute to its medical relevance [153]. Finally, research on the Philodryadini tribe, including *Philodryas* spp., *Chlorosoma* spp., and *Xenoxybelis* spp., uncovered significant intergeneric variability in venom composition, with each genus exhibiting distinct toxin profiles such as SVMPs, CRISPs, and CTLs, further underscoring the evolutionary divergence within this group [154]. Additionally, venom composition in newborns, female, and male adults was analyzed through transcriptomic data, which was further confirmed by proteomic data, revealing gender and ontogenetic variations in venom composition [155].

The cDNA sequencing approach of venomous tissues was also applied to venomous vertebrates other than snakes like fishes [156] and also to venomous invertebrates or their secretions [157,158,159], showing the power of this technology and providing information of medical importance, insights on venom evolution, biological activities of the venom and other aspects uncovered by the pioneering work of researchers from Instituto Butantan [138,139].

### 4.2. Toxin Gene Discovery and Venom Ontogeny

Transcriptomics plays a crucial role in the identification and detailed characterization of toxin genes, leveraging high-throughput technologies such as RNA sequencing (RNA-seq) to explore entire venom gland transcriptomes. This approach reveals a comprehensive inventory of both known and novel toxin genes [132,133], facilitating comparisons across diverse species and populations, and illuminating the genetic foundations of venom variation and the evolutionary pathways that have shaped its diversification [160]. Additionally, transcriptomics enables the quantification of toxin gene expression levels, offering profound insights into the relative abundance of various toxins within a venom sample [134]. This information is invaluable for understanding the functional roles of individual toxins and their cumulative contributions to the overall venom phenotype.

Furthermore, transcriptomics has significantly contributed to the discovery of novel toxin families and isoforms, expanding our understanding of venom complexity and diversity [136]. For example, the analysis of venom gland transcriptomes has uncovered previously unknown toxins with unique structures and functions, such as the multiple isoforms of svPLA_2_ enzymes in certain snake venoms, each exhibiting distinct substrate specificities and pharmacological activities [161], with far-reaching implications for drug discovery and development besides the understanding of their evolutionary trends in an ecological context [131,162].

In addition to identifying new toxins, transcriptomics has also proven essential in studying venom ontogeny, revealing how venom composition changes throughout a snake’s development [163]. By comparing gene expression profiles across different life stages, researchers have demonstrated how venom adapts to the evolving needs and ecological roles of snakes as they mature [135]. This knowledge is critical not only for understanding venom evolution but also for the development of age-specific antivenoms, as evidenced by landmark studies on the Viperidae snake *Bothrops jararaca* [155,164,165] and Elapidae monocled cobra (*Naja kaouthia*), which highlighted ontogenetic shifts in toxin composition [166]. Notably, studies on *B. jararaca* have shown significant differences in venom protein profiles and biological activities between newborn and adult snakes, with newborn venoms exhibiting distinct activities, such as lower proteolytic and hemorrhagic activity, but higher procoagulant and platelet aggregating functions, compared to adults [164]. In addition, ontogenetic shifts in the venom proteome complexity of *B. jararaca*, particularly in metalloproteinases, have been linked to changes in dietary habits as the species matures, with newborns preying on ectothermic animals and adults targeting endothermic prey [165]. These findings underscore the importance of incorporating venoms from different ontogenetic stages in antivenom production to enhance efficacy across age groups.

### 4.3. Current Transcriptomic Limitations and Ongoing Advancements

Although transcriptomics has led to significant advancements, certain limitations and complexities still need to be addressed. Transcriptomes are inherently dynamic, with variations influenced by factors such as age, diet, and environmental conditions, complicating the establishment of definitive venom profiles [50]. Additionally, while transcriptomics can identify the potential presence of toxins, it does not quantify their actual abundance or activity, necessitating proteomic analysis for confirmation and functional characterization. The complexity of venom gland transcriptomes demands sophisticated bioinformatics tools and considerable expertise for accurate data analysis and gene annotation, often requiring extensive experimental validation. Indeed, toxin scaffolds suggest potential biological activity, but these must be experimentally confirmed, as snake venoms result from the selection of new biological activities through the duplication and divergence of a few toxin scaffolds via mutations and accelerated evolution to acquire new functions, as indicated by genomic studies [167]. However, the limited number of high-quality snake genomes sequenced restricts our understanding of the evolution of snake toxins, venoms, and their regulation [168].

Nevertheless, ongoing advancements in sequencing technologies, bioinformatics algorithms, and functional assays are steadily improving our ability to address these challenges and unlocking new opportunities for deeper insights into venom biology. As these technologies continue to evolve, they will likely provide an even greater understanding of the genetic and molecular foundations of venom production, further enhancing the applications of transcriptomics in snake venom research and contributing to the development of novel therapeutic strategies.

## 5. Proteomics

Proteomics has emerged as a powerful tool for studying snake venoms, offering comprehensive insights into the complex protein mixtures that comprise these potent biochemical cocktails. Advanced proteomic techniques enable researchers to identify, quantify, and characterize numerous toxins within snake venoms, fostering a deeper understanding of their pharmacological effects and potential therapeutic applications [166,169,170,171,172]. This approach has revolutionized venom research, leading to the discovery of novel venom components and their mechanisms of action.

### 5.1. Mass-Spectrometry-Based Proteomics

Often combined with liquid chromatography (LC) methods like high-performance liquid chromatography (HPLC) [173] or ultra-performance liquid chromatography (UPLC) [174], mass spectrometry (MS) is a key technique in proteomics. It allows for the precise identification and quantification of proteins within complex mixtures. MS-based proteomics, usually involving tandem mass spectrometry (MS/MS or MS2) for peptide sequencing coupled with advanced software for protein database searches, has been instrumental in elucidating venom proteomes. It provides detailed maps of protein components and their post-translational modifications, enabling the detection of low-abundance proteins, often crucial for venom toxicity and variability [12,175,176].

### 5.2. Bottom-Up Proteomics and Early Challenges

Early proteomics studies primarily utilized bottom-up proteomics, a method from the 1990s. This involves enzymatically digesting proteins into smaller peptides, separating them using reverse-phase liquid chromatography (LC), and analyzing them with tandem mass spectrometry (MS/MS or MS2). While successful in handling complex mixtures, bottom-up proteomics produces an overwhelming number of peptides from complex protein samples, surpassing MS analytical capacity. To address this, two primary strategies for MS data acquisition were developed: data-dependent acquisition (DDA) [177] and data-independent acquisition (DIA) [178].

#### 5.2.1. Data-Dependent Acquisition (DDA)

Data-dependent acquisition (DDA) selects peptide precursors based on signal intensity during a precursor ion scan, fragmenting the most abundant ions to generate MS2 spectra, crucial for identifying peptides, characterizing post-translational modifications (PTMs), and providing structural insights. DDA is well-established and combining it with stable isotope labeling methods like Tandem Mass Tag Pro (TMTpro) significantly enhances sample throughput and quantification accuracy. TMTpro enables the concurrent analysis of multiple samples, delivering high-precision, comprehensive proteomic data. However, DDA undersamples, analyzing only a subset of available peptide precursors, resulting in uncharacterized portions of the proteome, potentially missing low-abundance peptides, and compromising the analysis’s comprehensiveness.

#### 5.2.2. Label-Free Quantification and Data-Independent Acquisition (DIA)

With the increasing number of samples in proteomics studies, especially in clinical cohort studies, single-cell proteomics, and systemic analyses, label-free quantification methods have gained favor over label-based approaches like TMTpro. Label-free methods face challenges, including missing values due to the stochastic selection of precursor peptides for fragmentation in narrow *m*/*z* isolation windows during DDA. Data-independent acquisition (DIA) offers a more robust alternative, enhancing protein identification by co-fragmenting all precursor ions within a preset *m*/*z* isolation window, covering the entire *m*/*z* range, and eliminating the stochastic nature of DDA. Research has shown that DIA can identify more proteins than the theoretical maximum number of MS2 spectra that an advanced high-resolution mass spectrometer can acquire using state-of-the-art DDA methods, demonstrating DIA’s superior comprehensiveness [179]. Additionally, multi-enzyme digestion strategies in proteomics have improved the identification of low-abundance toxins.

### 5.3. Top-Down Proteomics

While DDA, DIA, and multi-enzyme digestion approaches have advanced the field, they do not fully address the challenges inherent to bottom-up proteomics, which often results in the loss of critical information regarding intact protein structures, post-translational modifications, proteoform descriptions, protein interactions, and sequence variations. These data gaps hinder a comprehensive understanding of protein functionality and interactions, which is crucial for fully elucidating venom complexity.

By directly analyzing intact proteins without prior enzymatic digestion, top-down proteomics overcomes these restraints, as it enables the identification of full-length toxins and their isoforms, offering deeper insights into their mechanisms of action. A typical top-down proteomics workflow involves isolating intact proteins, followed by their separation using techniques like liquid chromatography (LC), and subsequent mass spectrometry (MS) analysis, where the intact protein ions are fragmented to provide detailed structural information. In light of this, top-down proteomics complements bottom-up methods, expanding our understanding of venom composition and driving advancements in therapeutic applications and antivenom development.

### 5.4. Innovations in Venom Proteomics

Top-down proteomics has proven to be a powerful tool for identifying venom proteoforms, offering a more detailed understanding of genetic variation, alternative splicing, single nucleotide polymorphisms (SNPs), and post-translational modifications (PTMs) compared to traditional bottom-up approaches [180]. This technique has been invaluable for comprehensive venom proteome analysis.

The future of snake venom proteomics likely involves comparative quantitative profiling across entire genera, which will provide deeper insights into venom evolution and potential medical applications [181]. A protein decomplexation strategy, combining in-solution trypsin digestion with mass spectrometry, has been developed for analyzing snake venom proteins, adding another layer of detail to proteomic analysis [182].

Innovative methodologies, such as high-throughput screening combined with venom nanofractionation and proteomics, have also been employed to identify snake venom toxins that affect coagulation, facilitating the development of advanced snakebite treatments [183]. Furthermore, hybrid elemental/molecular mass spectrometry setups in snake venomics have been demonstrated as a proof-of-concept for broader applications in proteomics, showing potential for more accurate quantification of venom components [12].

One important application of venom proteomics is antivenomics (Figure 1), which is a proteomic-based protocol used to quantify the cross-reactivity of antivenoms against homologous and heterologous venoms through immunoaffinity and immunodepletion protocols [39,184,185]. The combination of antivenomics and in vivo neutralization tests provides critical experimental data for the preclinical evaluation of antivenom efficacy.

### 5.5. Current Proteomics Limitations and Ongoing Advancements

Despite these advancements, snake venom proteomics still faces challenges, particularly in standardizing protocols for assessing antivenom efficacy. Standardized methods are crucial for reliably evaluating antivenom-neutralizing efficacy, as variability in toxicity assessment protocols across laboratories can lead to inconsistent results. Standardization would ensure more reliable and reproducible results, ultimately improving the accuracy of antivenom effectiveness and enhancing treatment outcomes for snakebite envenomation [186].

A comprehensive study of *Deinagkistrodon acutus* venom revealed a strong correlation between its protein composition and pharmacological effects. Proteomic analysis identified 103 proteins from 30 different snake venom families, with svPLA_2_, snaclec, antithrombin, thrombin, and metalloproteinases being the most abundant. This venom exhibits significant hematotoxic and neurotoxic effects, impacting the lungs and demonstrating potential anticoagulant and antithrombotic properties. However, the study emphasizes the need for enhanced methodologies to further elucidate these relationships and their implications [187].

The limited efficacy of hetero-specific antivenoms in cross-neutralizing procoagulant activities in Asiatic Mountain Pit Vipers highlights the need for improved antivenom development, especially given the high abundance of snake venom serine proteases contributing to their coagulotoxic effects. Enhanced antivenom formulations targeting these specific toxins are critical for treating envenomations caused by these species [188].

Challenges in antigenicity profiling have been identified, particularly in accurately detecting and characterizing low molecular weight toxins, which often exhibit poor immunogenicity. This necessitates improved techniques like advanced mass spectrometry and high-throughput screening to enhance binding efficacy and specificity, crucial for developing more effective antivenoms that comprehensively neutralize all venom components and improve clinical outcomes [189].

While Multi-Enzymatic Limited Digestion (MELD) has significantly improved toxin identification by increasing peptide overlap and enhancing downstream sequencing quality, it underscores snake venoms’ inherent complexity. This methodology identifies major toxins and uncovers less abundant proteins, highlighting the need for continuous methodological advancements to achieve comprehensive venom profiling [18]. Integrating ion mobility spectrometry (IMS) with liquid chromatography (LC) and mass spectrometry (MS) can enhance the separation and identification of venom peptides, particularly isomers and isobars, which may share the same mass but differ in structure.

## 6. Bioinformatics

Animal venoms hold significant untapped therapeutic potential, recognized not only for their toxicity but also for their extraordinary pharmacological diversity. As researchers continue to explore these complex biochemical mixtures, bioinformatics has emerged as a pivotal tool in venom research. Through advanced computational methods, bioinformatics enables scientists to analyze vast datasets, such as venom gland transcriptomes and proteomes, to uncover novel toxins and identify promising therapeutic candidates. By leveraging these technologies, researchers can accelerate the discovery and development of venom-derived drugs, targeting a range of different conditions from cardiovascular diseases [1,190] to cancer [191,192].

### 6.1. Computational Protein Modeling

#### 6.1.1. Traditional Methods

The three-dimensional architecture of venom proteins is closely linked to their function. Traditional computational methods like homology modeling and threading have laid the foundation for understanding the structural basis of venom toxicity. Homology modeling, which exploits structural similarities between target venom proteins and known templates, utilizes algorithms such as MODELLER [193] and Swiss-Model [194] to generate accurate structural models. Threading, which searches for compatible folds in protein structure databases using tools like I-TASSER [195] and RaptorX [196], has provided valuable insights into the structure–function relationships of diverse venom toxins, such as those found in snake venoms. These insights have fueled the design of antivenoms and paved the way for potential therapeutic interventions.

Homology modeling relies on the principle that proteins with similar sequences often adopt similar structures. By identifying a suitable template from the Protein Data Bank (PDB), researchers can build a model of the target venom protein using computational tools. Threading, on the other hand, does not require a close homolog but instead searches for compatible folds in protein structure databases. Both methods have been instrumental in elucidating the structure–function relationships of venom toxins, enabling the identification of key residues involved in toxicity and guiding the development of therapeutic agents.

For instance, homology modeling has been used to predict the structure of the svPLA_2_ enzyme from the venom of the Russell’s viper (*Daboia russelii*) [197]. This enzyme is responsible for the hemorrhagic effects of the venom, and its structure has been used to design inhibitors that can potentially be used as antivenom therapy. Threading has also been successful in predicting the structure of conotoxins, a class of peptide toxins found in the venom of cone snails [198]. These toxins have diverse pharmacological activities, and their structure-based design has led to the development of promising drug candidates for pain management and other therapeutic applications.

#### 6.1.2. Deep Learning in Protein Structure Determination

The advent of deep learning has introduced a transformative era for protein structure prediction. AlphaFold, developed by Google DeepMind, has demonstrated unprecedented accuracy in predicting protein structures directly from amino acid sequences, without the need for any previously known structure, a feat previously deemed intractable [199]. In venom research, this groundbreaking technology enables rapid and precise structural determination of uncharacterized venom proteins, accelerating the identification of novel therapeutic targets and drug candidates. A study by Jumper et al. showcased AlphaFold’s capabilities by predicting the structures of numerous proteins with high accuracy, including those from poorly studied organisms [28].

AlphaFold has significantly advanced venom research by accurately predicting the structures of more than 1000 snake venom toxins [31], being effective for proteins with disordered regions, such as loops and propeptides, emphasizing the benefits of tools like AlphaFold2 in understanding protein interactions, identifying binding sites, and designing molecules for diagnostics or treatments.

The ability to predict protein structures with high confidence holds immense promise for unlocking the therapeutic potential hidden within venoms. By rapidly and accurately determining the structures of venom proteins, researchers can gain a deeper understanding of their function, identify potential drug targets, and design novel therapeutics. Deep-learning-based protein structure prediction tools like AlphaFold are revolutionizing venom research and opening new avenues for drug discovery.

### 6.2. Virtual Screening of Venom-Derived Therapeutics

#### 6.2.1. Molecular Docking

Molecular docking simulations, computational methods that predict the preferred binding orientation of a ligand (e.g., venom toxin) to a receptor (e.g., ion channel), have become indispensable in venom research. By elucidating the molecular interactions between venom toxins and their targets, docking simulations not only predict toxicity but also guide the development of antivenoms and other therapeutic interventions. Docking software like AutoDock Vina [200] and GOLD [201] have been instrumental in identifying potential inhibitors of venom toxins.

Docking simulations involve predicting the binding pose of a ligand to a receptor and calculating the binding affinity. This information can be used to identify potential drug targets and design novel therapeutics. For example, docking simulations have been used to identify inhibitors of the svPLA_2_ enzyme from the venom of the Russell’s viper (*Daboia russelii*), responsible for the hemorrhagic effects of the venom [202]. These inhibitors can potentially be used as antivenom therapy.

In silico experiments offer a cost-effective and efficient means of exploring the vast chemical space of venoms. Another study by Chinnasamy et al. demonstrated this by discovering novel inhibitors of a snake venom metalloproteinase through virtual screening [203]. These inhibitors showed promising activity in preclinical studies and could potentially be developed into new drugs for the treatment of snakebite envenoming.

#### 6.2.2. Molecular Dynamics

Molecular dynamics simulations complement molecular docking by providing a dynamic view of the interactions between venom toxins and their receptors. By tracking the movement of atoms over time, these simulations reveal the conformational changes that occur upon binding, shedding light on the intricate mechanisms of venom toxicity [204,205]. Popular molecular dynamics software packages like GROMACS [206] and NAMD [207] have been employed to investigate the binding kinetics of venom toxins, the stability of toxin-receptor complexes, and the allosteric modulation of receptor activity.

Molecular dynamics (MD) simulations are powerful tools for investigating the dynamic behavior of venom toxins and their interactions with target receptors. By simulating molecular movements and interactions over time, MD simulations provide crucial insights into the functions and mechanisms of these toxins. For instance, MD simulations have been utilized to study the binding of alpha-cobratoxin to various receptors, revealing key residues involved in the interaction and the conformational changes that occur upon binding [208]. This knowledge is essential for developing effective therapeutic interventions, as it allows researchers to consider the conformational flexibility of toxins and design drugs that can specifically target and neutralize them.

Moreover, MD simulations have been instrumental in elucidating the mechanisms of various venom toxins. In a study by Preciado et al., MD simulations were used to investigate the molecular interactions between a snake venom metalloproteinase and its target protein, shedding light on the structural basis of its inhibitory activity. Such insights are crucial for developing novel therapeutic strategies to counteract the harmful effects of venom toxins [209].

#### 6.2.3. Databases and Tools

Snake venom research has significantly benefited from the advent of specialized bioinformatics databases, which curate extensive data on venom compositions, venomous species, and related molecular characteristics. These databases serve as essential repositories for researchers looking to understand venom diversity, toxin evolution, and therapeutic potentials.

One of the most comprehensive databases in this field is VenomKB (Venom Knowledge Base, https://github.com/JDRomano2/venomkb, accessed on 10 September 2024) [210], which compiles data from various studies on snake venom proteins and peptides, their sequences, structures, and functional annotations. This database provides detailed information on the toxic and non-toxic components of venom, facilitating the identification of novel bioactive molecules.

Another notable resource is Tox-Prot (https://www.uniprot.org/help/Toxins, accessed on 10 September 2024) [211,212], a specialized section within the UniProtKB/Swiss-Prot database, dedicated to venom proteins from various animal species, including snakes. Tox-Prot offers curated entries with rich annotations, including protein sequences, post-translational modifications, and functional information, essential for comparative venom studies.

VenomSeq (https://github.com/jdromano2/venomseq, accessed on 10 September 2024) is another valuable tool that focuses on the sequencing data of venom gland transcriptomes. It provides access to transcriptomic profiles of venom glands from numerous snake species, enabling researchers to study gene expression patterns and the evolution of venom components [213].

The ArachnoServer (https://arachnoserver.qfab.org/mainMenu.html, accessed on 10 September 2024) is primarily focused on spider venom but includes data on snake venoms as well [214]. It is particularly useful for researchers interested in the comparative analysis of venom across different venomous species.

Another recent effort emphasizes the integration of big data resources to improve venom research [215]. This framework underscores the importance of combining multi-omics data, such as genomics, transcriptomics, and proteomics, to uncover new insights into venom evolution and cross-species toxin functions. Such approaches align with the goals of existing databases by encouraging cross-disciplinary research and facilitating the exploration of therapeutic potentials in venom components.

Together, these databases and tools form the backbone of modern venom research, enabling deeper exploration of venom systems. The ongoing integration of big data resources [215] will further enhance our understanding of venom biology and its applications in biotechnology and medicine.

#### 6.2.4. Data Analysis and Interpretation

The analysis of sequences and structures of venom proteins is critical for understanding their functions and interactions. Several bioinformatics tools are widely used in snake venom research for this purpose.

BLAST (Basic Local Alignment Search Tool) [216], along with GenBank and UniProt, are well-established and indispensable database tools in the field. Researchers use BLAST to compare venom protein sequences against known sequences in these databases, as well as in specialized repositories like VenomKB [210], to identify homologous proteins and predict functions.

Clustal Omega [217] and MUSCLE [218] are powerful tools for multiple sequence alignment, which help in identifying conserved regions and evolutionary relationships among venom proteins. These tools are essential for phylogenetic analyses and for understanding the diversification of venom components.

Expasy ProtParam and ProtScale are tools available on the Expasy bioinformatics resource portal [219]. ProtParam allows for the computation of various physical and chemical parameters of proteins, such as molecular weight, isoelectric point, and amino acid composition. ProtScale helps in visualizing the hydrophobicity and hydrophilicity profiles of venom proteins, which are important for understanding their interaction with biological membranes.

For structural analysis, The PyMOL Molecular Graphics System (Schrödinger, LLC, New York, NY, USA) and ChimeraX [220] are widely used molecular visualization tools that allow researchers to visualize and manipulate 3D structures of venom proteins. ChimeraX is particularly noteworthy for its integration with AlphaFold [28], enabling researchers to directly determine protein structures within the application. These tools help in the identification of active sites and binding pockets and the design of inhibitors.

I-TASSER [195], used earlier for threading, also finds application in homology modeling, offering a comprehensive suite for structural predictions. Additionally, tools like MODELLER [193] and Swiss-Model [194] provide robust platforms for creating accurate 3D models based on known templates. These predicted models are crucial for understanding the structure–function relationships of venom components and for guiding experimental studies.

## 7. The Future of Venomics: Integrating Omics and Bioinformatics

In this review, we have thoroughly explored the transformative impact of modern technologies and bioinformatics tools on snake venom research. We examined the evolution of snake venom, emphasizing the intricate interplay of genetic and ecological factors that have shaped its remarkable diversity. We also discussed advancements in synthetic biology that have opened new avenues for understanding and utilizing venom components for therapeutic purposes. Transcriptomics and proteomics have revolutionized our ability to decipher the venom code, while bioinformatics has played a pivotal role in analyzing and interpreting the vast amounts of data generated by these omics technologies.

### 7.1. Multi-Omics Approaches

The future of venomics lies in integrating multiple omics technologies, including genomics, transcriptomics, proteomics, and metabolomics. This integrated approach, often referred to as venomics, allows for a comprehensive and holistic understanding of venom composition, function, and evolution. By combining data from different omics levels, researchers can gain insights into the genetic basis of venom production, the regulatory mechanisms controlling toxin expression, post-translational modifications of venom proteins, and interactions between venom components and their molecular targets. For instance, integrating transcriptomics and proteomics data can reveal how changes in gene expression translate into changes in protein abundance and activity. This information can identify key regulatory pathways and provide deeper insights into how venom composition is modulated in response to various stimuli.

One of the key advantages of multi-omics approaches is their ability to capture the dynamic nature of venom. Venom composition can vary significantly depending on factors such as the snake’s age, diet, geographical location, and environment. By integrating data from different time points and conditions, researchers can track changes in venom composition and identify the underlying factors driving these changes. This information is crucial for understanding the adaptability of venomous animals and their ability to fine-tune their venom in response to different ecological pressures. Take, for instance, the study by Modahl et al. [19], which used an integrated omics approach to investigate the regulatory networks controlling toxin gene expression in elapid and viperid snakes. The study revealed distinct regulatory mechanisms in these two snake families, highlighting the importance of considering evolutionary history in venom research.

Moreover, multi-omics approaches can help identify novel venom components with potential therapeutic applications. By combining proteomics data with transcriptomics and genomics data, researchers can identify and characterize previously unknown toxins with unique structures and functions. This can lead to the discovery of new drug leads and therapeutic targets for various diseases. An illustrative example is the recent study by Zheng et al. [20], which used a multi-omics approach to investigate the molecular mechanisms driving adaptation to diverse predator–prey ecosystems in closely related sea snakes. The study identified several novel toxins with potential applications in drug development.

Despite these advancements, a significant challenge remains in the functional characterization of the vast array of toxins identified through proteomics and transcriptomics. While proteomic and transcriptomic data provide sequences of these toxins, understanding their biological significance requires further functional assays and advanced bioinformatics analysis to predict and validate their targets and activities. This challenge is especially true for small peptide toxins, like three-finger toxins, whose diverse structural variations result in a broad spectrum of biological activities.

A crucial, yet often underexplored, component of venom research is metabolomics. While proteomics and transcriptomics focus on larger biomolecules such as proteins and RNAs, metabolomics investigates low-molecular-weight compounds such as amino acids, organic acids, alkaloids, and other small molecules that play pivotal roles in venom’s biological activity. Metabolomic profiling offers new insights into the full spectrum of bioactive compounds present in venoms, filling in critical gaps left by proteomic and transcriptomic studies. For instance, Klupczyńska et al. [221] highlighted the potential of metabolomics in venom research, particularly in revealing the presence of previously overlooked small molecules, such as bufadienolides and amino acids, which are essential for understanding venom complexity and its biological functions.

Metabolomics is especially powerful in its ability to characterize these smaller bioactive compounds, which may play crucial roles in venom’s evolutionary adaptability and interactions with prey or predators. By integrating metabolomics with genomics, transcriptomics, and proteomics, researchers can gain a more comprehensive understanding of how venom functions at different biological levels. For example, untargeted metabolomics can be used to profile the entirety of a venom sample, identifying unexpected molecules that contribute to venom’s efficacy [221]. Conversely, targeted approaches allow for the in-depth study of specific classes of small molecules. One such study by Pawlak et al. (2020) employed targeted metabolomics to analyze organic acids in honeybee venom, uncovering compounds that had previously been underappreciated in venom research [222].

The integration of metabolomics in venom research is critical not only for uncovering the full suite of bioactive compounds but also for understanding how venom composition varies across different species and environmental conditions. This could provide vital insights into venom evolution, enabling a deeper understanding of how venomous animals adapt to diverse ecological pressures. Additionally, the discovery of small molecules through metabolomics holds significant promise for the identification of novel drug leads, especially since many of these molecules have unique structures and functions not found in larger proteins. As such, metabolomics should be regarded as an essential complement to proteomics and transcriptomics in the study of venoms, bridging the gap between genotype and phenotype and offering a more complete picture of venom biology.

### 7.2. Bioinformatics in Venom Evolution

Bioinformatics plays a crucial role in analyzing and interpreting the vast amounts of data generated by multi-omics approaches. Advanced bioinformatics tools and algorithms are used to identify and annotate toxin genes, predict protein structures, model protein-protein interactions, and reconstruct evolutionary relationships. These tools are essential for understanding the molecular mechanisms of venom action, identifying potential drug targets, and designing novel therapeutics.

One of the key challenges in venom evolution studies is the identification of orthologous toxin genes across different species, which is essential for understanding the evolutionary history of venom toxins and their functional diversification. Bioinformatics tools, such as OrthoFinder [223] and ProteinOrtho [224], have been developed to facilitate the identification of orthologs in large-scale datasets. These tools use sophisticated algorithms to compare gene sequences and infer orthologous relationships based on sequence similarity and phylogenetic information.

Another challenge is reconstructing ancestral venom compositions by comparing those of living species and using phylogenetic information, allowing researchers to infer the profiles of ancestral species. This information provides insights into the evolutionary origins of venom and the selective pressures that have shaped its diversification. PAML [225] and HyPhy [226] are computational methods developed to estimate the evolutionary rates of toxin genes and determine the types of selection acting on them. These tools are crucial for identifying toxin genes that have undergone positive selection, a key indicator of adaptive evolution. By utilizing these methods, researchers can gain insights into the evolutionary processes shaping venom compositions, shedding light on the selective pressures and diversification events that have occurred over time.

### 7.3. Structure-Based Drug Design

Structure-based drug design (SBDD) is a powerful method for discovering new drugs by exploring the three-dimensional structure of target proteins. In venom research, SBDD has been used to design inhibitors of venom toxins, such as svPLA_2_ enzymes and metalloproteinases, which can potentially be used as antivenom therapy or as drugs for other therapeutic applications. One notable example is Varespladib [227], which was initially developed to treat inflammatory conditions such as acute coronary syndrome (ACS). Despite showing early promise, Varespladib was discontinued during Phase III clinical trials due to its failure to reduce cardiovascular events [228]. However, it has since been repurposed for envenomation therapy, where it effectively inhibits venom svPLA2 enzymes, demonstrating the potential of structure-based drug design (SBDD) in venom research [229].

The availability of high-resolution structures of venom toxins, determined by X-ray crystallography or nuclear magnetic resonance (NMR) spectroscopy, is essential for SBDD. However, not all venom toxins can be easily crystallized or studied by NMR. In such cases, computational protein modeling methods, such as homology modeling and threading, can be used to predict the structures of venom toxins. These predicted models can then be used for the virtual screening of small-molecule libraries to identify potential inhibitors. Several potential inhibitors of svPLA_2_ enzymes and metalloproteinases were identified in the study by Kalogeropoulos et al. [31], which utilized AlphaFold2 and ColabFold to predict the structures of several snake venom toxins and then employed these models for virtual screening of small-molecule libraries.

Once potential inhibitors are identified, they can be further optimized using computational methods such as molecular docking and molecular dynamics simulations. These approaches help refine the binding affinity and specificity of the inhibitors, as well as predict their pharmacokinetic and pharmacodynamic properties. For instance, molecular docking simulations have been used to predict the binding mode of Varespladib to svPLA_2_ enzymes, while molecular dynamics simulations have investigated the stability of the Varespladib-svPLA_2_ complex [230].

In addition, the recent application of Cryo-EM techniques has provided new insights into toxin–receptor/target interactions, enabling a better understanding of key interfaces involved in toxin activity and toxicity. Cryo-EM also helps elucidate the role of specific amino acids in neutralization by antibodies, offering new perspectives on structure–function relationships [23,24,25,26].

### 7.4. Venom-Derived Drug Development and Therapeutics

Venom-derived peptides and proteins have shown great promise as therapeutic agents for a wide range of diseases, including cancer, cardiovascular diseases, and neurological disorders. For example, captopril, an angiotensin-converting enzyme (ACE) inhibitor derived from the venom of the Brazilian pit viper (*Bothrops jararaca*), is a widely used drug for treating hypertension and heart failure [231].

The development of venom-based drugs is a complex and challenging process. A primary obstacle is identifying and isolating bioactive components from venom, which is a mixture of hundreds or even thousands of different molecules. Isolating the specific components responsible for therapeutic effects often requires a combination of chromatographic and spectroscopic techniques, along with functional assays to assess the bioactivity of the isolated compounds.

Another challenge is optimizing venom-derived peptides and proteins for therapeutic use. Venom components are often highly toxic and immunogenic, and they may need to be modified to reduce their toxicity and improve their pharmacokinetic properties. This can be achieved through various methods, such as chemical modification, protein engineering, and drug delivery systems. For example, researchers have used protein engineering to create modified versions of conotoxins with reduced toxicity and improved stability [232,233,234].

Despite these challenges, several venom-based drugs have been successfully developed and approved for clinical use [1,190,235]. These drugs have demonstrated significant therapeutic benefits for patients with various diseases, and they have the potential to revolutionize the treatment of many other conditions. The success of these drugs has encouraged further research into the therapeutic potential of venom, and we can expect to see even more venom-based drugs entering the market in the coming years.

### 7.5. Venom Bioprospecting and Synthetic Biology

Venom bioprospecting involves searching for new bioactive molecules in venom using methods like high-throughput screening, transcriptomics, and proteomics. This enables researchers to explore the chemical diversity of venoms, identifying compounds with unique biological activities. High-throughput screening rapidly tests thousands of venom fractions or compounds against specific targets, while transcriptomics and proteomics provide detailed profiles of gene expression and protein content, revealing insights into venom composition.

In this context, bioinformatics plays a crucial role in the identification and characterization of novel venom components by predicting the structures and functions of venom proteins, thus helping to understand their mechanisms of action. By integrating genomic, transcriptomic, and proteomic data, bioinformatics tools can prioritize candidates for experimental validation, including predicting protein interactions and modeling structures.

Complementing these approaches, synthetic biology, a rapidly growing field, designs and constructs new biological parts and systems. In venom research, synthetic biology is used to produce recombinant venom toxins and engineer venom components with improved therapeutic properties. For instance, recombinant versions of conotoxins, peptide toxins from cone snails, have been created through synthetic biology, showing promise as painkillers and other therapeutic agents.

Furthermore, synthetic biology offers the potential to create entirely new venom-inspired molecules with tailored properties. By modifying the genetic sequences that encode venom peptides, scientists can enhance stability, reduce toxicity, and improve pharmacokinetic profiles. This not only aids in developing better drugs but also reduces the need for venom extraction from animals, addressing ethical concerns.

The impact of these advancements is significant. Recombinant conotoxins have contributed to the development of novel analgesics that target specific pain pathways with high efficacy and fewer side effects compared to traditional medications [236]. Additionally, synthetic biology techniques have enabled the creation of venom-derived peptides with enhanced stability, making them more suitable for clinical use [237].

**Figure 3:**
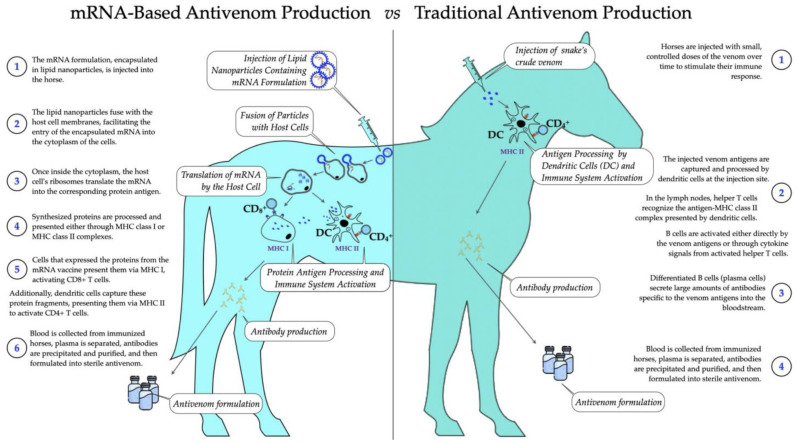
Production of snake antivenoms in horses. The traditional production using crude venom (**right side**) is compared with the potential production of antivenom using mRNA vaccines encoding toxins, formulated in lipid nanoparticles (**left side**).

Lessons learned from COVID-19 can also be applied to the production of specific antisera. While no studies have yet been conducted on the use of mRNA technology for this purpose, the concept is promising [238]. mRNA constructs based on toxin structures could be produced, formulated in lipid nanoparticles, and used as mRNA vaccines to immunize animals for the production of therapeutic hyperimmune antibodies directed against specific venom complexes, leveraging data from venomic studies (Figure 3).

## 8. Conclusions

The integrative omics and bioinformatics approaches discussed in this review have revolutionized our understanding of snake venoms, unveiling their complex molecular composition and evolutionary dynamics. The integration of genomics, transcriptomics, proteomics, and metabolomics, collectively known as “venomics,” has provided unprecedented insights into the genetic and regulatory mechanisms underlying venom production and diversification. These advancements have not only enhanced our knowledge of venom biology but also opened new avenues for therapeutic discovery and drug development.

As we look to the future, the continued evolution of omics technologies and bioinformatics tools promises to further unravel the complexities of venom systems. High-throughput sequencing and advanced mass spectrometry will continue to refine our understanding of venom composition, while deep learning algorithms and structural bioinformatics will enhance our ability to predict protein structures and interactions. Moreover, the application of synthetic biology will enable the production of venom components with tailored properties, facilitating the development of novel therapeutics with improved efficacy and safety profiles.

Despite these promising developments, challenges remain. The functional characterization of newly identified toxins, understanding the ecological and evolutionary drivers of venom diversity, and improving the specificity and efficacy of antivenoms are critical areas that require further research. Interdisciplinary collaborations and the integration of multi-omics data will be essential in addressing these challenges and translating basic research findings into clinical applications.

In conclusion, the future of venomics is bright, with the potential to significantly impact biomedical research and therapeutic innovation. By incorporating the power of integrated omics and bioinformatics approaches, we can continue to uncover the secrets of snake venoms and harness their potential for the benefit of human health. As our technological capabilities expand, so too will our understanding of these remarkable biological systems, paving the way for new discoveries and applications in the years to come.

## Data Availability

No new data were created or analyzed in this study. Data sharing is not applicable to this article.
